# Hazelnut Skin Fortification of Dehulled Lentil Chips to Improve Nutritional, Antioxidant, Sensory, and Chemical Properties

**DOI:** 10.3390/foods14040683

**Published:** 2025-02-17

**Authors:** Lara Costantini, Maria Teresa Frangipane, Riccardo Massantini, Stefania Garzoli, Nicolò Merendino

**Affiliations:** 1 Department of Ecological and Biological Sciences (DEB), Tuscia University, Largo dell’Università snc, 01100 Viterbo, Italy; 2 Department for Innovation in Biological, Agri-Food and Forest Systems (DIBAF), University of Tuscia, Via San Camillo de Lellis, 01100 Viterbo, Italy; 3 Department of Drug Chemistry and Technology, Sapienza University, 00185 Rome, Italy

**Keywords:** hazelnut cuticle, hazelnut pellicle, Tonda Gentile Romana hazelnut, pulses, legumes, food reformulation, palatability, sensory features, volatile composition, fibers

## Abstract

Legumes consumption is still low in Western countries, and their incorporation into bakery products could be a solution. However, a minimally processed legume-based product is still a challenge because of its negative impact on acceptance by consumers. Here, an oven-baked chip recipe, based on lentil flour, was fortified with 5% hazelnut skin (HS), a byproduct of hazelnut industrial processing, to improve the nutritional, antioxidant, and sensory features of this innovative food. Indeed, HS addition allows a nutritional profile improvement, increasing the fibers from 11.71% to 15.63%, and maintaining a high protein content (24.03 g/100 g). Furthermore, HS fortification increased total phenolic compounds and total antioxidant capacity by 1.6- and 2-fold, respectively, compared to the control. Finally, HS significantly improved the overall judgment score by 1.2 points (from 5.6 to 6.8 in control and experimental chips, respectively) halving the pulse-like aroma from 8.6 to 4.3 due to the strong decrease in the dodecane compound and due to HS volatile composition, rich in hexanal. Therefore, HS could be a valuable ingredient in improving the nutritional and functional features of bakery products as well as the sensory profiles of less palatable but healthy legume-based foods.

## 1. Introduction

Legumes are part of the botanical family *Leguminosae* or *Fabaceae* and indicate the whole pods and seeds (i.e., pulses). Increasing evidence from the literature suggests the potential of legumes as environmentally sustainable plant protein sources, with several health benefits for their high fiber and protein content as well as for the presence of essential minerals such as magnesium, potassium, iron, and zinc, B vitamins, and other bioactive compounds. Indeed, their steady consumption can lead to improved glycemia and reduce the cardiometabolic risk directly acting on blood pressure and heart rate but also on cardiometabolic biomarkers such as plasma total cholesterol, low-density lipoprotein-cholesterol, and apolipoprotein B levels [1]. For these reasons, legumes are suggested in the food-based dietary guidelines of several countries worldwide, although with different portion sizes based on the government [2]. Moreover, recent data showed a consumption below the dietary guidelines for legumes in an Italian population study of 10,916 adults [3]. Low legume consumption is also related to poor fiber consumption among Europeans (18 g/day) [4] in comparison to the suggested recommendation of the European Food Safety Authority (EFSA) (i.e., at least 25 g/day of fiber for adults) [5]. For all these reasons, in recent years, to improve legumes and fiber consumption in the population, several efforts have been made to create cereal-based foods enriched with pulse flours. Among these, lentils (*Lens culinaris*) are already used as flour in extruded and bakery products for their healthy nutritional features (i.e., protein content of 21–31%; carbohydrates content of 62–69%, out of which the majority is starch; and fiber 5–27%) and mild taste [6]. However, it is a challenge to formulate products with a high substitution of cereal flour with pulse flour, as it may negatively impact their texture and palatability and thus acceptance by consumers [1]. Moreover, considering the Westernization of the European diet with a significant consumption of ultra-processed foods rich in saturated fatty acids and simple sugars [7], the formulation of pulse flour-based products without the addition of food additives or the use of unhealthy cooking methods such as frying to improve their palatability, from the perspective of food reformulation [8], is an even greater challenge. In this context, food matrices discarded by the agri-food industries during the production phase can be innovative ingredients with peculiar sensorial features but also healthy characteristics due to their high content of fibers and antioxidants, which can be combined with pulse flours to produce innovative formulations. In a previous study, oven-baked wheat chips enriched with red lentil flour were formulated, and significant values for technological and sensory properties were described, although the maximum percentage of lentil flour used was 50% [9]. Here, we formulated oven-baked chip recipes exclusively based on lentil flour, and 5% hazelnut skin (HS) powder was added to improve the nutritional, antioxidant, and sensory characteristics of this innovative food. HS is a byproduct with a high content of fibers (50–70%), an antioxidant capacity about 42 times higher than the hazelnut fruit [10,11], and high content of monounsaturated oleic acid [12]. Moreover, beyond the significant nutritional and antioxidant features, the first evidence shows that HS from the Tonda Gentile Romana variety also has interesting sensory properties that are appreciated by potential consumers [13].

## 2. Materials and Methods

### 2.1. Ingredients and Chemicals

The hazelnut skins of the Tonda Gentile Romana (HSR) variety were sourced from a local producer, Bionocciola s.r.l. (Carbognano, Viterbo, Italy). After roasting the hazelnuts at 150 °C for 24 min, the skins were separated from the kernel and stored in vacuum-sealed plastic bags at 4 °C and kept in the dark. Dehulled lentils’ flour (DLF), extra-virgin olive oil (EVOO), salt, and water were bought from a local market. All chemicals were obtained from Merck KGaA (Darmstadt, Germany) unless stated otherwise.

### 2.2. Experimental Chips Recipe

**Figure 1:**
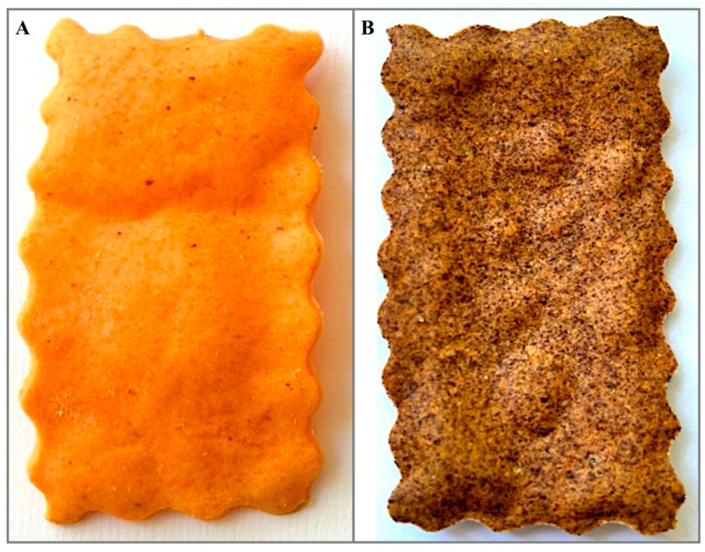
Experimental chips. (**Panel A**) CTRc, control chip; (**Panel B**) HSc5%, hazelnut skin chip 5%.

Mixtures of all the ingredients and HSR were prepared with a ratio of 5% (hazelnut skin chip 5%, HSc5%) or not (control chip, CTRc) of the total ingredient amount, as reported in Table 1. HSR was ground in a mill (Vevor, Shanghai, China) before being incorporated into the chips’ dough. All ingredients were combined in a mixer for 10 min. The resulting doughs were wrapped in polyethylene bags and allowed to rest at +4 °C for one hour. After resting, the doughs were manually rolled to 1 mm thickness and cut by pressing molds. The chips were baked in an oven at 170 °C for 20 min. Following a 30 min cooling period at room temperature, a portion of the chips was ground in a laboratory mill (IKA^®^ A11 basic Analytical mill (IKA^®^-Werke GmbH & CO., KG, Staufen im Breisgau, Germany). The resulting powders were stored in sealed polypropylene bags at −20 °C for later analysis. The powder was used for all analyses except the sensory evaluation. The experimental chips produced are shown in Figure 1.

### 2.3. Proximate Composition

The proximate composition was determined using two biological replicates for each sample, following the standard methods outlined by AOAC International (Association of Official Analysis Chemists International) [14]. Crude protein content (using a conversion factor of 6.25) was measured by the Kjeldahl method (AOAC 2001.11) using a SpeedDigester K-425 and Distillation Unit K-350 (BÜCHI, Labortechnik, AG, Uster, Switzerland). Crude fat (AOAC 920.39) was determined via Soxhlet extraction with petroleum ether as solvent, using a Soxhlet Extraction System B-811 (BÜCHI, Labortechnik, AG, Uster, Switzerland). Ash was determined by incinerating the sample in a muffle furnace at 550 °C for 4 h (AOAC 923.03). Total dietary fiber of all the experimental samples was measured using the enzymatic-gravimetric method (AOAC 991.43) (AOAC, 2006). Total carbohydrates were calculated by subtracting the sum of protein, fat, ash, and total dietary fiber (i.e., 100 − (g [protein + fat + ash + total dietary fiber] in 100 g of sample)). The energy value was calculated using the Atwater factor, as follows:

Energy value (Kcal) = (%Protein × 4) + (%Fat × 9) + (%Carbohydrate × 4) + (%Total dietary fibers × 2).

### 2.4. Extracts’ Preparation

For the analyses of the total phenolic compounds (TPC) and total antioxidant capacity (TAC), all samples were extracted following Costantini et al. 2014 [15], with three biological and two technical replicates processed and analyzed. In brief, the experimental powders of both chips’ samples were extracted overnight in the dark using 80% aqueous methanol (1:25, *w*/*v*). The samples were centrifuged at 10,000 rpm (ALC PK121R centrifuge; Bodanchimica s.r.l., Cagliari, Italy) for 10 min at 4 °C. The supernatants were collected and stored at −80 °C until further processing.

### 2.5. Total Phenolic Compounds (TPC) and Total Antioxidant Capacity (TAC)

TPC and TAC analyses were conducted as described in Farinon et al. 2024 [16]. Briefly, TPC was determined using the Folin–Ciocalteu method: 30 μL of deionized water was added to 10 μL of methanolic extract, 10 μL of Folin–Ciocalteu reagent, and 200 μL of 30% Na_2_CO_3_. After 30 min at room temperature, the absorbance of the mixture was measured at 725 nm. TAC was evaluated using two methods: ferric reducing antioxidant power (FRAP) and 2,2′-azino-bis (3-ethyl- benzothiazoline-6-sulfonic acid (ABTS^•+^) radical scavenging activity assays. For FRAP, 160 μL of FRAP assay solution (20 mM ferric chloride solution, 10 mM TPTZ solution, and 0.3 M acetate buffer) was mixed with 10 μL of the sample, standard, or blank and then dispensed into each well of a 96-well plate. Absorbance was measured at 595 nm at 37 °C after 30 min of incubation. ABTS^•+^ was assessed using the OxiSelect™ Trolox Equivalent Antioxidant Capacity (TEAC) Assay Kit (ABTS) (Cell Biolabs INC.) following the manufacturer’s instructions. TPC results were expressed as mg of gallic acid equivalents (GAE) per gram of dry weight (DW) of the sample. TAC determined by FRAP was expressed as mmol Fe^2+^ equivalents per gram of DW of the sample, while TAC measured by ABTS^•+^ was expressed as mmol of Trolox equivalents (TE) per gram of DW.

### 2.6. Sensory Analysis

The sensory analysis was conducted by eight official panelists, led by a panel leader, following the guidelines of UNI EN ISO13299 [17] in a sensory analysis-equipped laboratory according to ISO 8589 [18]. Two samples, CTRc and HSc5%, were evaluated by each panelist during each session. To minimize color differences between the samples caused by varying doughs, a red light was used during the evaluation. Panelists were instructed to evaluate both samples, with distilled water provided to cleanse their palates. A minimum 5 min break was taken between each sample. To ensure a common lexicon, three training sessions were conducted with the judges. After the training, a focus group determined the appropriate descriptors to use. Table 2 outlines the descriptors, definitions, and associated reference standards employed in the sensory analysis. Each descriptor was rated on an unstructured continuous scale, from 0 (absence of the characteristic) to 10 (maximum intensity), based on bibliographical sources [19,20]. The same scale was used to assess the descriptor of each panelist’s personal judgment, reflecting their subjective level of agreement. To better understand the differences in the sensory attributes and the overall rating between the samples, a principal component analysis (PCA) was performed.

### 2.7. HS-SPME/GC-MS Determination of Volatile Chemical Composition

The chemical volatile fraction of the samples was analyzed by using the SPME sampling technique. Approximately 0.5 g of each sample was placed into a 7 mL glass vial with a PTFE-coated silicone septum. Volatiles were extracted using SPME device from Supelco (Bellefonte, PA, USA) equipped with 1 cm fiber coated with 50/30 μm DVB/CAR/PDMS (divinylbenzene/carboxen/polydimethylsiloxane). Prior to use, the fiber was conditioned at 270 °C for 30 min. The equilibration time for all samples was obtained heating to 35 °C for 15 min. After this period, the fiber was exposed to the headspace of the samples for 50 min at 35 °C to capture and concentrate the volatile compounds. The SPME fiber was then inserted into the GC injector, maintained at 250 °C in splitless mode, for analyte desorption. The analyses were performed using a gas chromatograph coupled with a mass spectrometer Clarus 500 model Perkin Elmer (Waltham, MA, USA), equipped with a flame detector ionization (FID). A Varian Factor Four VF-5 capillary column was housed in the oven of GC. The programmed temperature applied started at 35 °C held for 1 min, then increased to 220 °C at 6 °C/min, and held for 15 min. Helium was used as the carrier gas at a constant flow rate of 1 mL/min. Components were identified by matching their mass spectra with those from the Wiley 2.2 and Nist 11 mass spectra libraries database and by calculating the linear retention indices (LRIs) using a series of alkane standards analyzed under the same conditions as the samples. The calculated LRIs were then compared with retention data from the literature. The peak areas of the FID signal were used to calculate the relative concentrations of the components, expressed as percentage, without the use of an internal standard or any correction factor. All analyses were performed in triplicate.

### 2.8. Statistical Analysis

The mean and standard deviation (SD) of the replicates were computed for all analyzed data. Statistical analysis was conducted using XLSTAT 2023.2.1414 (Addinsoft SARL, Paris, France) with a Student *t*-test. Differences were considered significant at the *p* < 0.05 or *p* < 0.01, as indicated. Principal component analysis (PCA) was used to gain a deeper understanding of how sensory descriptors influenced the overall evaluation of the chip samples.

## 3. Results and Discussion

### 3.1. Nutritional Evaluation of the Experimental Chips

The nutritional evaluation of the experimental chips for the protein, fat, total dietary fibers, ash, and energy determination is shown in Table 3. Lentils are among the typical pulses with the higher protein content after lupin and faba bean, with a 23–31% total protein content [21]. This value was confirmed in our experimental CTRc sample based on 70.20% DLF, where the total protein content was 24.03%. HSR possessed a protein content of 9.70% [13], and its 5% addition determined a modest but significant protein decrease in the HSc5% experimental sample, with a value of 23.09 g/100 g. Moreover, despite the HSR addition, the protein content found for HSc5% was similar to other 100% lentil fried snacks previously investigated in the literature where the protein content was 22.79% [22]. The fat content of lentil ranges from 2.6–2.8% [21]; in the CTRc experimental sample, fat content rose to 4.08% with the presence of EVOO in the recipe (Table 1). Moreover, the 5% HSR addition to the experimental chips determined a significant increase in total fat content from 4.08% to 6.68% due to the high-fat content of HSR (26.71%) [13]. Anyway, considering the oven-baking process, the increase in lipid content in these experimental samples is limited in comparison to the frying method, which determined a higher amount of lipids in the 100% lentil flour snacks (i.e., 17%) [22].

Consequently, due to the higher amount of lipids in HSc5%, a slight increase in total calories in HSc5% was found, 388.55 kcal/100 g, in comparison to 384.86 kcal/100 g in CTRc, although it should be considered that HSR lipid fractions are mostly constituted of monounsaturated oleic acid (i.e., 80.50%) [13]. However, despite the significant increase in lipid content in HSc5%, the caloric increase was slight due to the greater presence of HSR fibers. Indeed, it is known from the literature that lentils have variable fiber content ranging from 7 to 23% of total dietary fibers [21], and in the CTRc experimental sample, the contribution to the total dietary fiber nutritional component is due only to DLF, which determined an 11.71% total dietary fiber content (Table 3). Significant increase after HSR addition was found for the total dietary fibers (from 11.71% in CTRc to 15.63% in HSc5%) and ash (from 3.03% in CTRc to 3.39% in HSc5%) in consideration of the high value previously found for HSR (44.13% and 3.19% for total dietary fibers and ash, respectively) [23]. It should be noted that since the fiber content of HSc5% is more than 6% (15.63 g/100 g), this food may have the nutritional claim of “high fiber content” in Europe, as defined by the Regulation (EC) No 1924/2006 and lastly amended by Regulation (EU) No 1047/2012. In the literature, other strategies were applied to increase the fiber content of red lentil snacks. Among the most significant, the recent paper of Sinaki and Koksel analyzed the amount of total dietary fiber in extruded-lentil snacks 10–20% enriched with three dietary fiber sources (i.e., carrot powder, wheat bran, and red lentil hull) [24]. The higher total dietary fiber content was found for the 20% carrot powder-enriched lentil snack due to the higher total dietary fibers found in the carrot powder (45.99%) [24], comparable to HSR (44.13%). Therefore, considering the significant increase that was obtained with 5% HSR, greater increases in total dietary fiber could be obtained with higher percentages of HSR fortifications.

### 3.2. TPC and TAC of Experimental Chips

**Figure 2:**
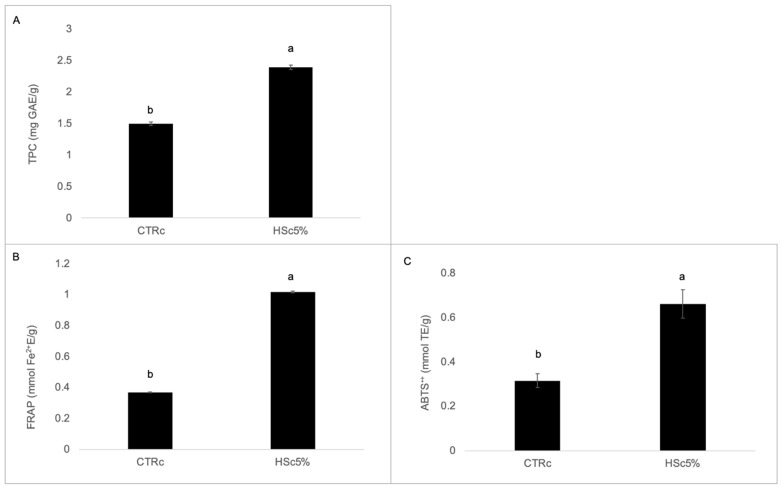
Total polyphenols content (TPC) and total antioxidant capacity (TAC) of experimental chips. (**A**) Total phenolic content (TPC) (mg GAE/g DW); (**B**) ferric reducing antioxidant power assay (FRAP) (mmol Fe^2+^/g DW); (**C**) ABTS^•+^ radical scavenging activity (mmol TE/g DW). Data represents mean ± standard deviation of n = 3 biological replicates and n = 2 technical replicates. Different letters indicate significant differences (*p* ≤ 0.05) according to one-way analysis of variance. CTRc: control chip; HSc5%: hazelnut skin chip 5%; GAE: gallic acid equivalent; TE: Trolox equivalent.

The TPC and TAC, determined with FRAP and ABTS^•+^ assays, of the experimental chips are shown in Figure 2. As already previously found by us, HSR addition determined a significant increase in the TPC and TAC in comparison to control recipes, with comparable values on same fortification percentages (i.e., 5% HSR), although in a different recipe (i.e., shortbread cookies) [23], and as previously demonstrated by other authors in different food matrices [25,26]. In particular, HSR addition determined a 1.6-times increase in the TPC of HSc5% in comparison to CTRc (1.50 ± 0.03 mg GAE/g in HSc5% in comparison to 2.39 ± 0.03 mg GAE/g in CTRc, Figure 2A) and more than a double increase in the TAC of HSc5% determined with both the assays in comparison to CTRc (1.02 ± 0.01 mmol Fe^2+^/g of HSc5% in comparison to 0.37 ± 0.00 mmol TE/g of CTRc in the FRAP assay, Figure 2B; 0.66 ± 0.06 mmol TE/g of HSc5% in comparison to 0.32 ± 0.03 mmol TE/g of CTRc in the ABTS^•+^ assay, Figure 2C).

For TPC and TAC, the comparison with the literature was difficult due different recipes, extraction methods, and applied analytical methods. For TPC, although similar values were found also in the paper of Babacan Cevik et al. (1.02–1.18 mg GAE/g) [9], it should be considered that the recipe in this study provided for a 50% maximum percentage of lentil flour combined with a “base flour” prepared by mixing wheat flour (80%), corn flour (20%), onion powder (2%), garlic powder (2%), and cumin (2%) [9]. Higher values were found for TPC in the papers of Lv et al. (3.83 mg GAE/g), even if on extruded snacks based on 49% whole lentil flour (i.e., with seed coats) and 49% potato starch, but they were also obtained with different extraction method [27]. For TAC, the comparison is even more complicated considering that in these studies, the DPPH method was used, and the results were compared to different standard curves [9,27].

### 3.3. Sensory Analysis of the Experimental Chips

In the sensory analysis, HSc5% obtained the highest score in the overall judgment of the panel compared to the CTRc (median values 6.844 and 5.611, respectively). The two products showed significant differences (*p* < 0.05) for all sensory descriptors (Table 4). It is particularly noteworthy that in HSc5%, the presence of some detected attributes, such as roasty, coffee-like, walnut aroma, hazelnut aroma, and caramel aroma, were instead completely absent in the CTRc product. This result marked a deep sensory difference between the two formulated chips. Our results also show that the HSc5% chip had half the pulse-like and potato attributes of the CTRc product (4.333 vs. 8.667 and 4.389 vs. 7.333, respectively) and is therefore more balanced and pleasant tasting.

**Figure 3:**
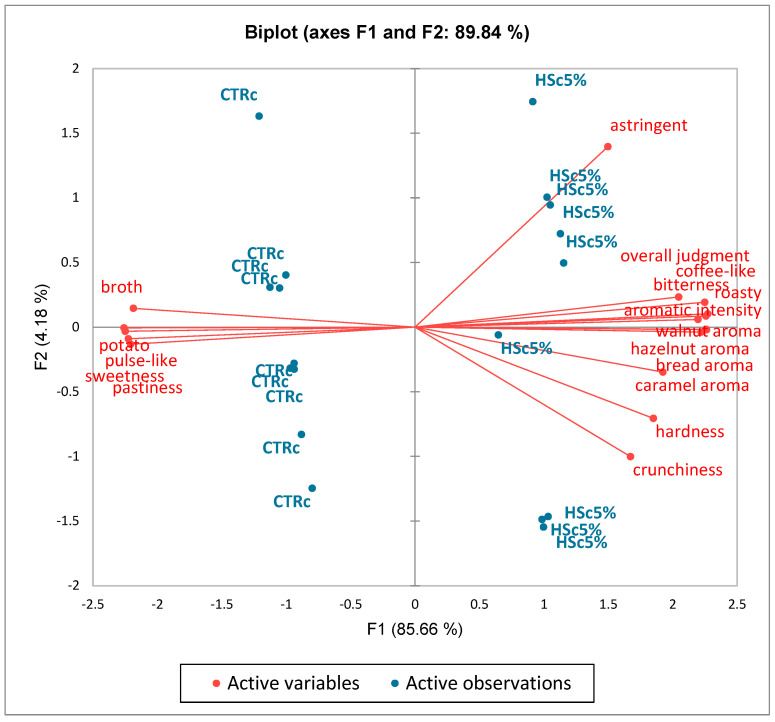
PCA loading plot showing the multivariate variation between the two products in terms of sensory attributes and overall judgment. CTRc: control chip recipe; HSc5%: hazelnut skin chip 5% recipe.

Regarding the PCA evaluation, Figure 3 shows the clear separation of the two samples along F1, with the HSc5% chip on the positive side of F1 and the CTRc chip on the negative side. In all, 89.84% of the total variance is explained by the first two components (85.66% F1 and 4.18% F2). The attributes, overall judgment, aromatic intensity, coffee-like, roasty, walnut aroma, caramel aroma, hardness, crunchiness, and hazelnut aroma had the values that contributed most to F1. These descriptors were related to HSc5%, whereas the attributes of broth, pulse-like, potato sweetness, and pastiness were closely associated with the CTRc sample. This is evident from the PCA biplot, which shows a clustering of HSc5% in the first and second quadrants, while the CTRc is mainly in the third and fourth quadrants. Therefore, the difference in sensory characteristics between the two products under consideration was confirmed. Finally, in sensory analysis, the performance of the panel is of paramount importance, as it is indispensable for reliable and certain results. Indeed, it is the panelists’ judgment that determines the outcome of the evaluation.

Table 5 shows the control of assessor performances. The left side is a summary of the descriptors, which are sorted by their product discrimination. All attributes were discriminated by the panel; that is, there are differences between the two products for each attribute under consideration. In the same table, the assessors are ranked according to their average rank of the individual effects on all attributes. If the panelist discriminated between the two products (*p* < 0.05) and agreed with the panel, + is shown; otherwise, − is shown. Checking panelists’ performance showed excellent results for most attributes: all panelists discriminated between the two products and agreed with the rest of the panel (+ cells). For the walnut aroma, four panelists (numbers 1, 4, 5, and 9) were discriminant and disagreed with the rest of the panel (− cells). Panelist no. 2 was the only one who detected differences between the products by pulse-likeness and disagreed with the panel (− cell). Also, for the hazelnut aroma, panelists number 1, 5, and 9 were discriminant and disagreed with the panel. The ranking of the panel members based on RankF shows that panel member no. 7 was the most discriminating when all the attributes were considered, while panel member no. 1 was the least discriminating. Our findings highlighted that overall, the experts had a good individual agreement with the whole panel.

Here, for the first time, the HSR fortification was analyzed to improve the sensorial analysis of snacks based on lentil flour. Indeed, in previous papers, it was shown that snacks based on lentil flour achieved a substantially low flavor acceptability score, indicating that flavor was the greatest challenge to the development of a successful pulse-containing snack [28,29]. To the best of our knowledge, in the literature, only one paper applied another strategy to improve the flavor in pulse-based snacks in a way comparable with the present study. Indeed, Sattar et al. fortified “diamond cuts” snacks with 9% germinated pulse flour and compared them with 9% non-germinated pulse flour. This strategy improved the overall acceptability score by 1.2 points (from 6.2 to 7.4 in non-germinated vs. germinated pulse flour, respectively); additionally, the maximum percentage of incorporated pulses was low and equal to 9% [30]. Here, similarly, HSR significantly improved the overall judgment score by 1.2 points (from 5.6 to 6.8 in CTRc and HSc5%, respectively), with the pulse-like aroma decreasing from 8.6 to 4.3 but with 65.2% DLF in the recipe.

### 3.4. Volatile Chemical Compositions of the Experimental Chips

Considering the significant differences between samples obtained from the sensorial analysis described in the Section 3.3, analysis of the volatile chemical composition was performed to describe the HSR aromatic ability. By HS-SPME-GC/MS analysis, thirty-one compounds were detected and identified in total, and the obtained results are shown in Table 6. Specifically, 21 analytes were found in both samples but with qualitative and quantitative differences. Indeed, some compounds, such as hexane, 2,4-dimethyl—(0.8%), hexanal (7.2%), octane, 3,3-dimethyl—(7.3%), octane, 2,4,6-trimethyl—(9.2%), nonanal (20.1%), L-camphor (1.3%), nonane, 2,2,4,4,6,8,8-heptamethyl—(2.0%), β-copaene (0.5%), 2-undecanethiol, 2-methyl—(3.6%), and *γ*-muurolene (0.8%), were present only in the HSc5%. On the other hand, some aliphatic hydrocarbons, such as 2,4-dimethyl-1-heptane (0.1%), decane (0.9%), nonane, 2,6-dimethyl—(1.1%), undecane (1.3%), 3-pinanylamine (0.3%), 5-ethyldecane (0.1%), undecane, 5-methyl—(0.1%), 2,3-dimethyldecane (0.7%), undecane, 3-methyl—(1.2%), and hexadecane (0.7%) were detected only in the CTRc (Table 6).

From a quantitative point of view, the most relevant differences concerned the following compounds: heptane, 2,4-dimethyl- reached 11% in the HSc5% compared to 0.1% in the CTRc; octane, 3,5-dimethyl- was 3.8% in the HSc5% compared to 1% of CTRc; farnesan was 3.9% in the HSc5% compared to 0.5% of CTRc; and finally, dodecane was predominantly present in the CTRc (87.4%) compared to the HSc5% (14.1%). In particular, the strong decrease in the dodecane compound in HSc5% in comparison to CTRc, having a gasoline-like odor [31], could be the main factor responsible for the improvement in the sensory characteristics previously described by sensory analysis (Section 3.3). Nonanal and hexanal, characterizing the HSc5%, are aldehyde compounds responsible for a characteristic aroma. Nonanal has a strong citrus odor that gives a floral note added in perfumes, while hexanal is used in the flavor industry to produce fruity flavors. To better understand which ingredient was responsible for the emission of metabolites by the two samples, we performed the analysis using the same technique also on DLF and HSR. The obtained results listed in Table 7 highlight that the two monoterpene compounds limonene and 1,8-cineole are released by lentil flour, and in particular, limonene was the compound found with the higher percentage, in accordance with a previous study [31,32].

It should be considered that cooking affects the pulses’ volatile composition [33], and for this reason, a direct correlation between the obtained data from the experimental chips (Table 6) and the raw DLF (Table 7) was not found. Instead, to the best of our knowledge, this is the first study to investigate the HS volatile composition. A previous study investigated the volatilome composition of raw and roasted whole Tonda Gentile Romana hazelnuts [34]; however, several differences were reported in comparison to our study. Indeed, while the main compound detected in HSR was hexanal (Table 7), which is characterized by fatty and green-leafy notes, in the paper of Stilo et al., the main compounds found in the whole roasted Tonda Gentile Romana hazelnut were 3-methylbutanal and 2,3-pentanedione, with malty and buttery odor quality, respectively [34]. This could indicate that HS has a characteristic volatile composition that is distinct from that of the whole hazelnut. However, further studies are needed to confirm this hypothesis.

## 4. Conclusions

In conclusion, here, we showed that addition of hazelnut skin from the Romana variety to a dehulled lentil-based snack allows a nutritional profile improvement, increasing the fiber content by 33.5% and maintaining a high protein content (24.03 g/100 g), in addition to a significant TPC and TAC increase in comparison to control (1.6- and 2-times increase, respectively). Furthermore, hazelnut skin from the Romana variety can improve the sensory profile of the lentil-fortified chips, attenuating pulse-like and potato attributes and earning the fortified snack a higher overall judgment score by the official sensorial panelists (6.844 of the fortified snack in comparison to 5.611 of the control, on a 0–10 scale). The volatile chemical composition analysis revealed a strong decrease in the dodecane compound, with a gasoline-like odor, in HSc5% compared to CTRc. Finally, for the first time, the hazelnut skin volatile composition was investigated, highlighting its peculiar volatile chemical profile, which differs from previous data reported in the literature relating to whole Romana hazelnuts. Therefore, hazelnut skin could be a valuable ingredient in improving the nutritional and functional features of bakery products as well as the sensory profiles of less palatable but healthy legume-based foods. It is worth noting that using hazelnut skin as a by-product of the industrial processing of hazelnuts plays an important role in environmental sustainability and the global agricultural economy.

## Figures and Tables

**Table 1:** Recipes of experimental and control chips.

Recipe	Control Chip (CTRc)	Hazelnut Skin Chip 5% (HSc5%)
Dehulled lentil flour (DLF)	70.2%	65.2%
Hazelnut skin Romana (HSR)	-	5.0%
Water	26.3%	26.3%
Extra-virgin olive oil (EVOO)	2.9%	2.9%
Salt	0.6%	0.6%

**Table 2:** Descriptors used for sensory assessment and related reference standards.

Descriptors	Sensory Attribute Definitions	Standards and Reference Materials
Hardness	Force required to compress the sample with the back teeth	Ripe banana and raw carrot
Crunchiness	Amount of noise generated when the sample is chewed at a fast rate with the back teeth	Value 0 corresponds to a dried apple piece, while 10 corresponds to a fresh celery piece
Pastiness	Amount of soft, smooth mass that does not release moisture during chewing	Watermelon and peanut butter
Astringent	Sensation of drying, drawing-up or puckering of any of the mouth surfaces	Diluted tannic acid solution (0.06–2 mg/mL)
Sweetness	Basic taste associated with sugar (sucrose)	Diluted sucrose solution (0.5–6 g/L)
Bitterness	Basic taste associated with caffeine	Diluted caffeine solution (0.03–0.2 g/L)
Broth	Aromatics associated with boiled meat, soup or stock	1 stock cube in 2 L of bolt water
Pulse-like	Basic taste associated with pulses	Lentil seeds in hot water (1:3 (*w*/*v*))
Hazelnut aroma	Intensity of aroma of hazelnut products	Taste of hazelnut
Caramel aroma	Aromatics associated with caramel	Taste of caramel
Walnut aroma	Aromatics associated with walnuts	Taste of walnut
Bread aroma	a general term used to describe the aromatics in the Flavor associated with uncooked grains such as corn, oats and wheat	Ground mixture of rice flour, white flour, yellow cornmeal and oatmeal
Potato	Aromatics associated with cooked potato	Potato cooked during 5 min in boiling water
Coffee-like	Aromatics associated with coffee-like	Taste of coffee
Roasty	Aromatics associated with roasty	Taste of roasted

**Table 3:** Proximate composition of experimental and control chips (g/100 g).

	Protein ^1^	Fat	Carbohydrate ^2^	Total Dietary Fibers	Ash	kcal/100 g	kJ/100 g
*CTRc*	24.03 ± 0.11 ^a^	4.08 ± 0.27 ^b^	57.15	11.71 ± 0.46 ^b^	3.03 ± 0.02 ^b^	384.86	1610.24
*HSc5%*	23.09 ± 0.21 ^b^	6.68 ± 0.04 ^a^	51.21	15.63 ± 0.05 ^a^	3.39 ± 0.05 ^a^	388.55	1625.71

Data are means ± standard deviation of two (n = 2) replicates. Different letters indicate significant differences (*p* ≤ 0.05), according to Student’s *t*-test. CTRc: control chip recipe; HSc5%: hazelnut skin chip 5% recipe. ^1^ Conversion factor: 6.25. ^2^ As difference (i.e., 100—(g [protein + fat + total dietary fiber + ash] in 100 g of dry weight sample)).

**Table 4:** Least squares means of sensory attributes and overall judgment of the two products.

Attributes	HSc5%	CTRc	Pr > F(Model)	Significant
Hardness	7.889 ^a^	6.667 ^b^	0.000	Yes
Crunchiness	8.722 ^a^	8.000 ^b^	0.003	Yes
Pastiness	0.900 ^b^	2.044 ^a^	<0.0001	Yes
Sweetness	0.833 ^b^	2.833 ^a^	<0.0001	Yes
Bitterness	3.778 ^a^	0.944 ^b^	<0.0001	Yes
Astringent	0.278 ^a^	0.000 ^b^	0.006	Yes
Broth	2.000 ^b^	3.000 ^a^	<0.0001	Yes
Pulse-like	4.333 ^b^	8.667 ^a^	<0.0001	Yes
Hazelnut aroma	2.650 ^a^	0.300 ^b^	<0.0001	Yes
Caramel aroma	0.389 ^a^	0.000 ^b^	<0.0001	Yes
Walnut aroma	2.056 ^a^	0.222 ^b^	<0.0001	Yes
Bread aroma	4.000 ^a^	2.667 ^b^	<0.0001	Yes
Potato	4.389 ^b^	7.333 ^a^	<0.0001	Yes
Coffee-like	2.722 ^a^	0.000 ^b^	<0.0001	Yes
Roasty	6.667 ^a^	0.000 ^b^	<0.0001	Yes
Aromatic intensity	6.944 ^a^	6.056 ^b^	<0.0001	Yes
Overall judgment	6.844 ^a^	5.611 ^b^	<0.0001	Yes

Means that show different letters are significantly different (*p* ≤ 0.05) according to Student’s *t*-test. CTRc: control chip recipe; HSc5%: hazelnut skin chip 5% recipe.

**Table 5:** Control of Assessor performance in the sensorial analysis.

Attributes Summary:					Assessors Summary:					
Attributes	FProd	7	2	5	9	8	6	3	4	1
Roasty	273,780	+	+	+	+	+	+	+	+	+
Coffee-like	136,765.5	+	+	+	+	+	+	+	+	+
Potato	104,458.2	+	+	+	+	+	+	+	+	+
Aromatic intensity	34,105.6	+	+	+	+	+	+	+	+	+
Sweetness	30,976	+	+	+	+	+	+	+	+	+
Bitterness	29,443.81	+	+	+	+	+	+	+	+	+
Broth	28,302.4	+	+	+	+	+	+	+	+	+
Hardness	26,208.8	+	+	+	+	+	+	+	+	+
Bread aroma	12,742.23	+	+	+	+	+	+	+	+	+
Walnut aroma	8428.423	+	+	−	−	=	+	=	−	−
Pulse-like	7765.902	+	−	+	+	+	+	+	+	+
Overall judgment	6240.364	+	+	+	+	+	+	+	+	+
Astringent	4344.897	+	+	+	+	+	+	=	+	+
Caramel aroma	2621.44	+	+	+	+	+	+	+	+	=
Hazelnut aroma	2353.613	+	+	−	−	+	+	+	+	−
Pastiness	1178.133	+	+	+	+	+	+	+	+	+
Crunchiness	833.333	+	+	+	+	+	+	+	+	+
RankF		4	4.206	4.324	4.588	5.353	5.471	5.529	5.588	5.941

FProd: Fisher statistics of the product effect; RankF: average of the ranks of the product effects by assessor on all descriptors; the assessors and descriptors are sorted according to their discriminating power (RANKF and FPROD, respectively); +: discriminating assessor and in agreement with the panel; −: discriminating and disagreeing assessor; =: non-discriminating assessor.

**Table 6:** Volatile chemical composition (percentage mean value ± standard deviation) of CTRc and HSc5%, as determined by HS-SPME/GC–MS.

N°	COMPONENT ^1^	LRI ^2^	LRI ^3^	CTRc (%)	HSc5% (%)
1	hexane, 2,4-dimethyl-	731	728	-	0.8 ± 0.06
2	heptane, 4-methyl-	768	764	0.5 ± 0.02 ^b^	3.5 ± 0.04 ^a^
3	hexanal	807	804	-	7.2 ± 0.08
4	heptane, 2,4-dimethyl-	818	821	0.1 ± 0.01 ^b^	11.0 ± 0.10 ^a^
5	2,4-dimethyl-1-heptene	831	836	1.6 ± 0.03	-
6	octane, 3,5-dimethyl-	925	928	1.0 ± 0.03 ^b^	3.8 ± 0.05 ^a^
7	octane, 3,3-dimethyl-	930	935	-	7.3 ± 0.06
8	*α*-pinene	937	941	0.1 ± 0.01 ^b^	1.6 ± 0.04 ^a^
9	heptane, 4-propyl-	941	945	0.2 ± 0.02 ^b^	1.4 ± 0.05 ^a^
10	octane, 2,4,6-trimethyl-	953	955	-	9.2 ± 0.3
11	decane	1001	1000	0.9 ± 0.04	-
12	nonane, 2,6-dimethyl-	1018	1025	1.1 ± 0.04	-
13	limonene	1033	1031	0.4 ± 0.02 ^b^	1.9 ± 0.02 ^a^
14	1,8-cineole	1035	1033	0.3 ± 0.02 ^b^	3.1 ± 0.02 ^a^
15	nonanal	1110	1105	-	20.1 ± 0.08
16	undecane	1118	1115	1.3 ± 0.04	-
17	decane, 2,4,6-trimethyl-	1124	1121	0.4 ± 0.02 ^b^	1.3 ± 0.03 ^a^
18	L-camphor	1142	1139	-	1.3 ± 0.04
19	3-pinanylamine	1145	1142	0.3 ± 0.02	-
20	5-ethyldecane	1148	1146	0.1 ± 0.01	-
21	undecane, 5-methyl-	1155	1156	0.1 ± 0.01	-
22	2,3-dimethyldecane	1161	1158	0.7 ± 0.02	-
23	undecane, 3-methyl-	1173	1171	1.2 ± 0.03	-
24	dodecane	1216	1214	87.4 ± 10.12 ^a^	14.1 ± 0.08 ^b^
25	dodecane, 2,6,11-trimethyl-	1325	1320	0.8 ± 0.02 ^b^	1.5 ± 0.02 ^a^
26	nonane, 2,2,4,4,6,8,8-heptamethyl-	1331	1329	-	2.0 ± 0.04
27	farnesan	1386	1381	0.5 ± 0.02 ^b^	3.9 ± 0.05 ^a^
28	*β*-copaene	1431	1426	-	0.5 ± 0.02
29	2-undecanethiol, 2-methyl-	1435	1433	-	3.6 ± 0.05
30	*γ*-muurolene	1491	1486	-	0.8 ± 0.02
31	hexadecane	1615	1612	0.7 ± 0.02	-
	SUM			98.7	99.8

^1^ The components are reported according to their elution order on apolar column; ^2^ linear retention indices measured on polar column; ^3^ linear retention indices from the literature; data are means ± standard deviation of three (n = 3) replicates. Means with different letters, in the same row, are significant different according to Student’s *t*-test (*p* < 0.01). CTRc, control chip; HSc5%, hazelnut skin 5% chip.

**Table 7:** Volatile composition (percentage mean value ± standard deviation) of lentil flour and hazelnut film, as determined by HS-SPME/GC–MS.

N°	COMPONENT ^1^	LRI ^2^	LRI ^3^	DLF (%)	HSR (%)
1	hexanal	810	812	-	61.4 ± 1.10
2	2,2,5,5-tetramethylhexane	865	870	1.9 ± 0.03	-
3	3-butenyl pentyl ether	978	982	-	1.7 ± 0.03
4	heptane, 2,2,4,6,6-pentamethyl-	981	985	-	5.8 ± 0.05
5	*cis-β*-ocimene	1020	1024	11.5 ± 0.12	-
6	limonene	1028	1031	22.1 ± 0.14	-
7	1,8-cineole	1030	1033	7.5 ± 0.08	-
8	*trans-β*-ocimene	1035	1034	7.0 ± 0.03	-
9	*β*-terpinene	1037	1036	2.1 ± 0.08	-
10	*n*-propyl heptyl ether	1080	1075	-	27.3 ± 0.25
11	2,6,8-trimethyl-decane	1110	1104	4.0 ± 0.03	-
12	isothujol	1115	1110	-	1.7 ± 0.07
13	1,3,8-p-menthatriene	1120	1118	3.3 ± 0.02	-
14	2,5,9-trimethyldecane	1122	1121	4.2 ± 0.04	-
15	2,4,6-trimethyldecane	1124	1122	11.5 ± 0.08 ^a^	2.0 ± 0.02 ^b^
16	2,5,9-trimethyldecane	1126	1123	3.8 ± 0.04	-
17	pentanoic acid, hydrazide	1159	1161	2.0 ± 0.02	-
18	6-ethyl-2-methyldecane	1191	1185	4.3 ± 0.03	-
20	ylangene	1380	1376	4.7 ± 0.02	-
21	2,6,10,14-tetramethylheptadecane	1876	1872	9.9 ± 0.07	-
	SUM			99.8	99.9

^1^ The components are reported according to their elution order on apolar column; ^2^ linear retention indices measured on polar column; ^3^ linear retention indices from the literature; data are means ± standard deviation of three (n = 3) replicates. Means with different letters, in the same row, are significant different according to Student’s *t*-test (*p* < 0.01). DLF, dehulled lentil flour; HSR, hazelnut skin Romana.

## Data Availability

The original contributions presented in this study are included in the article. Further inquiries can be directed to the corresponding author.
